# An Epidemiological Analysis of Head Injuries in Taiwan

**DOI:** 10.3390/ijerph15112457

**Published:** 2018-11-04

**Authors:** I-Lin Hsu, Chung-Yi Li, Da-Chen Chu, Li-Chien Chien

**Affiliations:** 1 Department of Surgery, College of Medicine, National Cheng Kung University Hospital, National Cheng Kung University, Tainan 70403, Taiwan; yilinhsu@gmail.com; 2 Department of Public Health, College of Medicine, National Cheng Kung University, Tainan 70403, Taiwan; cyli99@mail.ncku.edu.tw; 3 Department of Public Health, College of Public Health, China Medical University, Taichung 40402, Taiwan; 4 Department of Neurosurgery, Taipei City Hospital, Taipei 10065, Taiwan; DAD57@tpech.gov.tw; 5 Department of Emergency Medicine, Taipei City Hospital, Taipei 10065, Taiwan

**Keywords:** head injury, incidence, national health insurance, population-based study, mortality, traumatic brain injury

## Abstract

Traumatic head injuries occur frequently in Taiwan, having catastrophic consequences for the victims, their families, and society as a whole. However, little is known about the risk factors at the population level in Taiwan. The primary aim of this study was to obtain more information on these variables and their relationships. Another aim was to analyze the effects of independent variables such as sex, age, residency, pre-existing conditions, mechanisms of injury, associated injuries, and severity on the probability of in-hospital death. Using the 2007–2008 total admissions claim dataset from Taiwan’s National Health Insurance system, total admissions due to acute head injury were selected for further analysis. The obtained data included patient demographics and trauma hospitalization rate. A total of 99,391 patients were admitted with head injury, 48,792 of which had moderate-to-severe head injury. There were 4935 cases recorded as in-hospital mortality and the standardized in-hospital mortality rate was 10.7 deaths per 100,000 person-years. The mortality rate increased with age. After adjustments, male sex, age older than 54 years, living in a rural area, lower monthly income, a Charlson comorbidity index greater than one, being a pedestrian hit by a motor vehicle, fall from a height, and having significant chest, abdominal, or lower extremity injury increased the risk of death during admission. This population-based analysis provides information about the incidence rate and death rate for admissions in Taiwan due to acute head injury and the factors that affect in-hospital mortality. Our results that highlight the risk factors for adverse outcome can help us prevent or improve rural area trauma care of head injury patients in the future.

## 1. Introduction

Trauma is a devastating cause of morbidity and mortality in Taiwan and other countries around the world. Head injury is still one of the leading causes of death in modern society [1,2,3]. Approximately 1.5 million patients are suffering from traumatic head injuries in the United States [4] and traumatic head injuries caused more than 53,000 deaths every year during 1997 to 2007, at a rate of 18.4 deaths per 100,000 people [5]. Critical head injuries are preventable deaths. Head injuries lead to tremendous loss of capacity and resources in long-term care. Some studies have noted the severity of social burden caused by head injuries and claimed that more than half of trauma-related deaths are caused by head injury [6,7]. Evidence has accumulated from studies in other regions indicating that head injury is a global health threat [8,9,10,11]. Some epidemiological reports have revealed various incidence rates in different countries [7,12,13,14,15,16,17,18]. Comparison amongst studies is difficult because the definitions of head injury, socio-economic status, and the inclusion criteria are different. A descriptive, population-based study of patients with severe associated head injuries has not been carried out in Taiwan. Therefore, our aim was to estimate the trauma hospitalization rate in Taiwan due to head injury and describe where people are being treated for their injuries. We also aimed to investigate the prevalence of concomitant injuries among hospitalized patients with acute head injury in Taiwan, and how hospital outcomes are affected by variables including (1) patient characteristics including age, sex, residence, and presence of pre-existing conditions (PECs) and (2) characteristics of the injury including body region of principal injury, trauma severity, mechanism of the injury. There are two major events affecting trauma case mortality rate: the introduction of the motorcycle helmet law in 1997 and the new trauma care hospital classification system in 2009. The helmet law effectively decreased the mortality and morbidity from motorcycle-related head injuries after its inception [19]. Even in rural areas, people now wear helmets when riding their motorcycle leading to a decrease in accident mortality rate. We wanted to find more predictors associated with mortality of head injury allowing policy makers to introduce new policies or improve medical care, thus decreasing the loss.

## 2. Methods

In Taiwan, the National Health Insurance (NHI) system covers nearly 99% of the population and this study retrieved the entire claim admissions dataset for those who were coded with discharged diagnostic numbers defined by the International Classification of Diseases, 9th revision, Clinical Modification (ICD-9-CM). Therefore, this study included all NHI trauma patients who were hospitalized and discharged between 1 January 2007 and 31 December 2008 and is population-based. We captured all the trauma hospitalizations of patients defined as any admission claim coded with at least one ICD-9-CM diagnosis between 800.00 and 959.99, except 905.00 to 909.99, which indicate “late effects of injuries, poisonings, toxic effects and other external causes: 930.00 to 939.99, which include “effects of a foreign body entering through an orifice”, and 958, “complications”. In Taiwan, computed tomography (CT) for patients with traumatic head injury is generally covered by NHI and moderate-to-severe head injury was mainly diagnosed by radiological findings on CT, as in other studies [13]. This research is a based on the national data bank with informed consent. This research did not involve human participants and/or animals.

### 2.1. Variables

According to capacity and services, hospitals in Taiwan were classified into three levels: medical centers (MC), regional hospitals (RH), and local hospitals (LH). Taiwan’s definition for medical centers and regional hospitals includes capacity and services. Empirically, the patients were stratified into nine age groups: 0–14, 15–24, 25–34, 35–44, 45–54, 55–64, 65–74, 75–84, and >84 years of age. The patient area of residency was classified into two groups: urban (including suburban) and rural areas. The average population density (persons/km^2^) is higher in urban areas (2635 persons/km^2^) than in rural areas (230 persons/km^2^). According to the level of monthly income, injured patients were classified into four groups: monthly income less than USD $660, between USD $660 and $1320, more than USD $1320, and dependents, which are those who were injured but not employed, including those insured (NHI) by a spouse, other family member, or social welfare. During the study period, the average Taiwan monthly living cost was USD $595/person. Pre-existing conditions were defined as the medical co-morbidities of the injured patients who were diagnosed and recorded with ICD-9-CM codes in the same admission data file [18]. We used Charlson’s comorbidity index to quantify the PECs [20]. According to Charlson’s study in 1987, 17 diseases are included in the formula for calculating the Charlson comorbidity index [21]. The patients were classified into four levels according to their Charlson comorbidity index: 0, 1, 2, and more than 2.

For assessing injury severity, a computerized mapping method that used the ICD codes to obtain injury severity scores, such as the AIS, was employed [22,23]. A computerized mapping system, ICDMAP, for converting injury-related International Classification of Diseases, ninth revision (ICD-9-CM) rubrics into AIS scores, was proposed by MacKenzie et al. in 1989, and their results have been verified [24]. This ICD mapping system has been refined over the years and has been used in several large or population-based studies to classify severity using ICD diagnostic codes [25,26]. Our study applied ICDMAP to the dataset and derived ICD/AIS scores for each injury diagnosis and an ICD/ISS (injury severity score) for each admission.

As the patient group coded with an AIS head score of 1 or 2, as generated by ICDMAP software, was classified as mild head injury, we defined moderate-to-severe head injury as significant head injury with an AIS head score more than 2, which included moderate (AIS head = 3) and severe head injury (AIS head 4, 5, and 6), as shown in Table 1.

ICD E-codes in the claim data were also used to classify the mechanism of injury [27]. A patient is classified as the driver or passenger in a motor vehicle traffic accident if the number after the decimal point is zero or one, respectively (e.g., E811.0, E811.1, E812.0, or E812.1). A patient is classified as a motorcycle rider or passenger injured in a traffic accident if the number after the decimal is two or three, respectively (e.g., E811.2, E811.3, E812.2, or E812.3). A patient is classified as having an injury of an unspecified nature in a motor vehicle traffic accident if the number after the decimal point is nine (e.g., E811.9, E812.9, etc.), as shown in Table 2.

### 2.2. Statistical Analysis

The percentage of sex, four categories of monthly income, four categories of PECs, and mortality rates were compared to understand the differences between all patients with head injuries and the patients with moderate-to-severe head injuries. Means, medians, and standard deviations of continuous variables, such as age, monthly income, Charlson comorbidity index, and ICD/ISS were analyzed using analysis of variance (ANOVA). The relative odds of death in the admission course were estimated by using binary logistic regression with the backward conditional stepwise method. Statistical significance was set at *p* < 0.05. All the data analyses were generated by using SPSS software, version 20.0 (IBM Corporation, New York, NY, USA). Institutional board review was waived because this study involved secondary data analysis of a well-encrypted database.

## 3. Results

### 3.1. Incidence of Hospitalization and In-Hospital Fatality Rate

**Figure 1:**
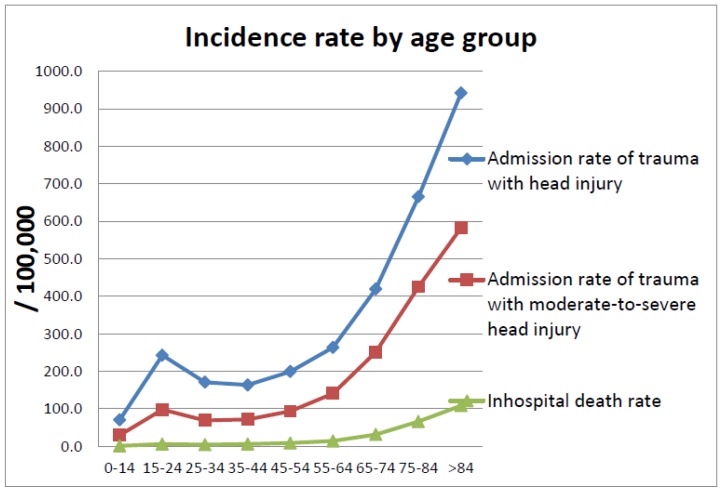
Incidence rate of head injury admission and death by age groups.

During 2007–2008, 476,241 cases were retrieved as trauma admissions. There were 50,599 cases of mild head injury in which the AIS in the head region was 1 or 2. Among these cases, 151 deaths occurred and the in-hospital mortality rate was 0.3%. There were 9666 cases of moderate head injury (AIS head of 3) and 126 deaths were recognized; the in-hospital mortality rate was 1.3%. There were 39,126 cases of severe head injury (AIS head of 4, 5, or 6) and 4809 deaths were recognized; the in-hospital mortality rate was 12.3%. The raw hospitalization rate for all head injuries in Taiwan was 215.3 per 100,000, and for moderate-to-severe head injury, 105.9 per 100,000. After calculations using the U.S. 2000 standard population, the standardized incidence of all head injury was 220.6 per 100,000 in Taiwan and the standardized incidence of moderate-to-severe head injury was 110.5 per 100,000. Table 3 shows the incidence of trauma hospitalization with head injury in the nine age strata. The lowest trauma hospitalization rate for patients with moderate-to-severe head injury in Taiwan was in the 0–14 year age group (29.8/100,000), followed by a higher incidence in the 15–24 year age group (97.9/199,999), and gradually increased up to 582.4/100,000 in the age group older than 84 years. The graph pattern of hospitalization rate in the nine age strata and the in-hospital fatality rate for patients with moderate-to-severe head injury are shown in Figure 1. The in-hospital mortality rate for patients with moderate-to-severe head injury was 10.7% and the standardized hospitalization death rate was 11.8/100,000. The hospitalization death rate for patients with moderate-to-severe head injury increased gradually from 1.2/100,000 in the group younger than 15 years old to the highest rate, 108.5/100,000, in the group older than 84 years, except for slightly lower rates in the 25–34 year age group than in the 15–24 year age group. Men had higher rates than women in all age groups.

**Figure 2:**
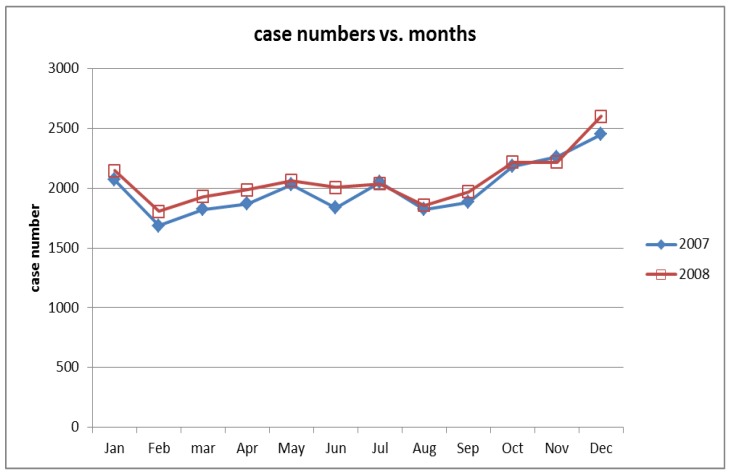
Monthly distribution of case numbers during 2007–2008.

In Taiwan, 6,935,205 people resided in rural areas in 2008. During the study period, 41,570 rural people were admitted with head injury and 18,108 of these rural people had moderate-to severe head injury. Thus, focusing on moderate-to-severe head injury, the patients who resided in rural areas had a higher hospitalization rate than those residing in urban areas (130.6 vs. 95.3 per 100,000, respectively; *p* < 0.01). Among the patients who had in-hospital mortality, 2038 were registered in rural areas. The rural population yielded a higher raw hospitalization death rate for patients with moderate-to-severe head injury than the urban population (14.2 vs. 9.2 per 100,000, respectively; *p* = 0.005). Monthly distribution of case numbers during 2007–2008 is shown in Figure 2, and in general, the occurrence did not significantly change with month.

**Figure 3:**
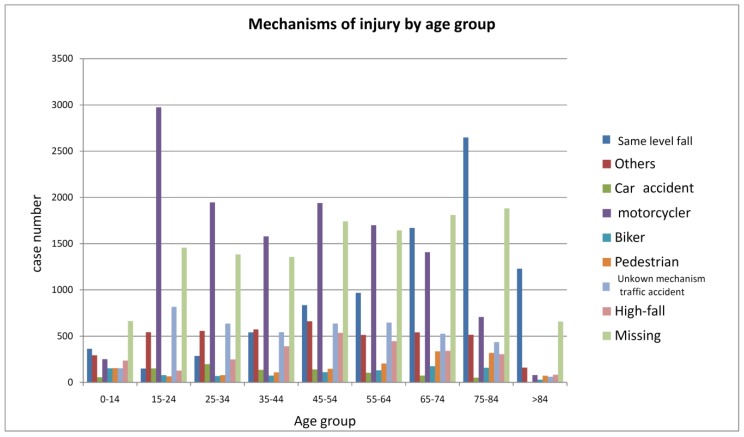
Mechanisms of head injury by age groups.

Table 4 presents the demographics of two groups: all patients with head injury and those with moderate-to-severe head injury. Table 2 summarizes the comparisons of age, sex, and other demographics, the associated injury characteristics, and distribution of the locations of treatment between these two groups. The patients with moderate-to-severe injury were older (50.6 ± 23.1 vs. 46.8 ± 22.9 years), a higher proportion were men, and rural people had a higher Charlson comorbidity index and a higher ICDISS score (18.2 ± 6.4 vs. 12.0 ± 8.1 years), and had a longer median stay in the hospital (7, 4–13 vs. 5, 2–6 (days, 25th–75th centile). In the moderate-to-severe head injury group, more patients were treated at a medical center and fewer at a local hospital. Figure 3 shows the different distribution of the mechanisms of injury among these nine age groups. Among those in the younger population, being injured as a motorcycle rider or passenger was more frequently the external cause, and a fall on the same level was more frequently the cause in older patients. The proportion of case numbers for motorcycle injury in people aged 15–24 years was significantly higher than that in people of other ages (49.4% vs. 26.4%, *p* < 0.001).

### 3.2. Results of Regression Analyses

We included characteristics of the patients and their injuries, including age, sex, rural residency, level of monthly income, Charlson comorbidity index, mechanism of injury, and ICD/ISS in a logistic regression to estimate the adjusted relative odds of in-hospital fatality for the patients with moderate-to-severe head injury. The results are shown in Table 5. Different age strata experienced different effects; being younger than 15 years decreases the risk of death, but being older than 54 years is associated with a higher risk of death. The older the patient, the higher the hospitalization mortality rate. Rural residency increases the risk of death (odds ratio (OR): 1.19, 95% confidence interval (CI): 1.11–1.27). Compared with the lowest level of monthly income (less than USD $660), the group with a monthly income between USD $660 and 1320 had a lower probability of death (OR: 0.78, 95% CI: 0.72–0.85), but dependents had a higher probability of in-hospital death (OR: 1.49, 95% CI: 1.38–1.61) Compared with the patients treated at medical centers, being treated at a regional hospital was associated with a higher probability of in-hospital fatality (OR: 1.11, 95% CI: 1.03–1.19). Compared with the patients treated at private hospitals, being treated at a public hospital was associated with slightly lower risk of death (OR: 0.92, 95% CI: 0.86–1.00).

As an indicator of PECs, higher Charlson comorbidity index scores were associated with a higher risk of death. The relative odds of dying in the group with a Charlson comorbidity index of two was 1.52 times higher than those for the group with a Charlson comorbidity index of zero (95% CI: 1.34–1.72) and the relative odds of dying in the group with a Charlson comorbidity index more than two was 2.5 times higher (95% CI: 2.16–2.90). Several mechanisms of injury did not significantly affect the probability of death. Compared with falls on the same level, only pedestrians hit by a motor vehicle and falls from a height had a higher probability of in-hospital death. Furthermore, being associated with another significant injury significantly increased the risk of mortality; the mortality rate in the group associated with a significant abdominal, chest, or lower extremity injury was 3.41, 3.41, and 1.37 times higher, respectively, than the group without a significant injury.

## 4. Discussion

Head injury continues to be a major health problem around the world in both developed and developing countries [1,2,3,4]. The aim of this study was to examine the incidence and in-hospital head injury-related mortality in Taiwan. Compared with other regions or countries, the incidence rate in our study, 220.6/100,000, was higher than in the U.S. (103/100,000), Finland (101/100,000), and India (160/100,000) but lower than in Europe (235/100,000) [2,9,16,28]. However, the definitions and inclusion criteria are different, which complicates comparisons between studies and regions.

Among those aged 15 to 74 years, the most common mechanism of injury is motorcycle-related traffic accident because motorcycles are the most popular transportation vehicles in Taiwan. Motorcycle riders and passengers should be the target population for injury prevention, even in the age group between 65 to 74 years. For those older than 65 years, falls caused more than two-thirds of the injuries and hospitalization rates in this age group. This deserves more attention because trauma does not only affect the younger population; therefore, a focus on injury prevention programs should be aggressively advocated for the elderly. For the people younger than 15 years old in Taiwan in the study by Tsai et al. in 2004, the incidence rate was higher in the age groups of 4 to 9 and 10 to 14 years. The main cause of pediatric head injury was traffic injury followed by falls. Of all pediatric traffic injuries, motorcycle-related injury had the highest incidence, followed by pedestrian and bicycle-related injury [29].

In the United States, head injuries cause about 2.5 million emergency department (ED) visits, 280,000 hospitalizations, and 50,000 deaths annually [4]. In Taiwan, our study showed a hospitalization death rate of 11.8/100,000. Compared with the head injury-related death rates in the U.S. (18.1/100,000) [18], Finland (18.3/100,000) [28], India (20.0/100,000), and Europe (15.4/100,000) [22], the standardized death rate in Taiwan, at 11.8/100,000, was lower than in these other studies. However, our dataset only included patients who had been admitted to hospital, and head injury-related accidents caused 4935 in-hospital deaths among the total 7640 hospitalization mortality cases in 2007–2008 in Taiwan. The dataset did not include deaths at the scene of the accident, during transportation, or in the emergency department. In Taiwan, there were 14,207 accidental deaths in 2007–2008 and our in-hospital dataset only included half of the deaths. It is reasonable to assume that the annual head injury-related mortality rate is underestimated in this study. With the aging population, the devastating effects of head injury will become increasingly serious.

Our regression model showed that only pedestrians hit by a motor vehicle and those who had a fall from a height were associated with a higher probability of mortality; other causes of head injury were not. This result might be caused by the lack of helmet protection and pedestrians hit by a motor vehicle is a well-known dangerous mechanism of injury [30]. In Taiwan, NHI provides a high accessibility to hospital resources to all citizens, but our results show that rural residents and the subgroup with lower monthly income were associated with lower survival probability after moderate-to-severe head trauma. Some researchers have found that the medical institutions providing higher levels of trauma care are often located in urban areas [31]. Their results showed that the odds ratio for death was higher in the hospitals located in rural areas than in hospitals located in more urbanized areas. One study found huge rural-urban disparities in mortality from unintentional injuries [32]. Lower monthly incoming and living in a rural area are associated with many factors leading to higher mortality rate, such as low education and health literacy, risky environments, and low economic support for medical care. We need to focus on these vulnerable populations, reinforce cause-specific prevention programs to reduce the mortality rate, relocate more resources to fairly provide optimal care, and periodically monitor the effectiveness of these programs.

This study had some limitations. First, this is a retrospective study including all the NHI claim data about injury-related hospitalizations during the study period. However, the accuracy of NHI claim data is assured given the severe penalty for fraud and erroneous claiming. Second, this dataset does not include the Glasgow Coma Scale (GCS), which may be misleading for assessing the severity of head injury. The NHI covers brain CT for head injury in an emergency setting, and the severity score ICD/ISS in this study was basically determined by brain CT. The third limitation is that nearly 10% of all admissions were recorded as traffic accidents without further information about the mechanisms, and one-quarter of admissions were recorded without any information about the mechanism of injury. This is reasonable because there was a higher proportion of trauma patients who were found injured without any witnesses or other information source compared to in the United States [14]. Because this study is based on the NHI research database, some detailed information that was not included may lead to ignoring some clinical important factors such as alcohol or drug consumption, related to head injury mortality.

## 5. Conclusions

In conclusion, this epidemiological study that focused on traumatic head injury hospitalizations in Taiwan is the first population-based study providing abundant objective information about the severity of the damage caused by head injury in our society. Although the government and medical institutes have attempted to mitigate the damage caused by head injury for decades, head injury still requires public attention to alleviate the impact on human health and society. Our results that highlight the risk factors for adverse outcome can help us to prevent or improve rural area trauma care for head injury patients in the future.

## Figures and Tables

**Table 1:** Abbreviated Injury Score (AIS) head score and head injury severity classification.

AIS Head Score	Head Injury Severity Classification
AIS score 1–2	Mild
AIS score 3	Moderate
AIS score 4–6	Severe

**Table 2:** ICD-E codes and injury mechanisms.

ICD-E Code	Injury Mechanism
The number after the decimal point is zero or one, e.g., E811.0	Driver or passenger in a motor vehicle traffic accident
The number after the decimal point is two or three, e.g., E811.2	Motorcycle rider or passenger injured in a traffic accident
The number after the decimal point is nine, e.g., E811.9	An injury of an unspecified nature in a motor vehicle traffic accident

ICD-E code: International Classification of Disease-external cause of injury code.

**Table 3:** Age distribution of trauma hospitalizations with head injury in Taiwan in 2007–2008.

Age	Total	Hospitalizations with Head Injury	% of Total Admission with Head Injury	Moderate-to-Severe Head Injury	Population in Taiwan, 2008	Admission Rate of Trauma with Moderate-to-Severe Head Injury (per 100,000)	In-hospital Deaths with Moderate-to-Severe Head Injury	In-hospital Death Rate in Age Group (per 100,000)
0–14	27,764	5497	5.5%	2324	3,905,203	29.8	90	1.2
15–24	61,981	15,778	15.9%	6363	3,248,257	97.9	383	5.9
25–34	61,409	13,368	13.4%	5404	3,911,237	69.1	342	4.4
35–44	59,961	12,099	12.2%	5302	3,702,101	71.6	452	6.1
45–54	72,028	14,405	14.5%	6751	3,611,743	93.5	633	8.8
55–64	59,074	11,901	12.0%	6358	2,256,270	140.9	642	14.2
65–74	55,950	11,506	11.6%	6886	1,372,060	250.9	859	31.3
75–84	56,108	10,990	11.1%	7026	825,992	425.3	1091	66.0
>84	21,966	3847	3.9%	2378	204,168	582.4	443	108.5
Total	476,241	99,391	100.0%	48,792	23,037,031	105.9	4935	10.7

**Table 4:** Patients demographics in mild and moderate-to-severe head injury cases.

Patient Characteristics	Total Head Injury Cases	Moderate-to-Severe Head Injury Cases	*p*-Value	
Variable	*N* = 99,391	*N* = 48,792 (49.1%)		
Age (years)				
Mean (± SD)	46.8 ± 22.9	50.6 ± 23.1	<0.001	
>64 years old	26,343 (26.5%)	16,290 (33.4%)	<0.001	
Sex				
Male	61,746 (62.1%)	32,072 (65.7%)	<0.001	
Residency				
Rural	41,570 (41.8%)	18,108 (37.1%)	<0.001	
Income				
Income group (dependent)	30,673 (30.9%)	14,793 (30.7%)	<0.001	
Income group (<USD $660)	28,071 (28.2%)	14,257 (29.6%)		
Income group (USD $660–1320)	35,719 (35.9%)	16,792 (34.8%)		
Income group (>USD $1320)	4918 (4.9%)	2404 (5.0%)		
Charlson Index groups				
Charlson index = 0	81,865 (82.4%)	38,159 (78.2%)	<0.001	
Charlson index = 1	12,551 (12.6%)	7233 (14.8%)		
Charlson index = 2	3317 (3.3%)	2231 (4.6%)		
Charlson index > 2	1658 (1.7%)	1169 (2.4%)		
Mechanism of Injury				
Car accidents	2785 (2.8%)	917 (1.9%)	<0.001	
Motorcycle riders or passengers	29,930 (30.1%)	12,586 (25.8%)		
Bicycle	2219 (2.4%)	981 (1.9%)		
Pedestrians (hit by motor vehicle)	2751 (2.8%)	1488 (3.0%)		
Traffic accident, mechanism unknown	8722 (8.8%)	4456 (9.1%)		
Same level fall	7072 (14.0%)	8694 (17.8%)		
Fall from a height	15,705 (4.9%)	2716 (5.6%)		
Missing	20,767 (20.9%)	12,598 (25.8%)		
Associated injury (ICD/ISS ≥ 3)				
Face injury	28	12	0.279	
Neck injury	21	13	0.573	
Chest injury	3397 (3.4%)	2298 (4.7%)	<0.001	
Abdominal and pelvic injury	1017 (1.0%)	531 (1.1%)	0.047	
Spinal injury (Thoraco-lumbar spine)	1591 (1.6%)	658 (1.3%)	<0.001	
Upper extremity injury	444 (0.4%)	171 (0.4%)	<0.001	
Lower extremity injury	3099 (3.1%)	1674 (3.4%)	<0.001	
ICDISS				
Mean	12.0 ± 8.1	18.2 ± 6.4	<0.001	
Level of treating hospitals				
Medical Center (MC)	20,546 (20.7%)	16,611 (34.0%)	<0.001	
Regional Hospitals	46,258 (46.5%)	23,354 (47.9%)		
Local Hospitals	32,587 (32.8%)	8827 (18.1%)		
Ownership of treating hospitals				
Public Hospitals	22,439 (22.6%)	10,795 (22.1%)	0.001	
Treatment Outcome				
Discharge Status				
Death	5086(5.1%)	4935 (10.1%)	<0.001	
Length of Stay (day)				
Mean	7.8	10.7	<0.001	
Median	5	7		
25th–75th centile	2–6	4–13		

**Table 5:** Regression results. Adjusted odds of in-hospital death for the patients with moderate-to-severe head injury.

Vairables	Relative Odds	Lower 95% CI	Higher 95% CI	*p*-Value
Men (Women as reference)	1.18	1.10	1.26	<0.001
Age (15–54 years old (y/o) as reference)				
Child (<15 y/o)	0.53	0.42	0.66	<0.001
55–64 y/o	1.43	1.29	1.58	<0.001
65–74 y/o	1.77	1.62	1.95	<0.001
>74 y/o	2.38	2.19	2.59	<0.001
Residency (urban as reference)				
Rural resident	1.19	1.11	1.27	<0.001
Income group (<USD $660 USD as reference)				
Income group (dependent)	1.49	1.38	1.61	<0.001
Income group (USD $660–1320)	0.78	0.72	0.85	<0.001
Income group (>USD $1320)	0.97	0.82	1.14	0.717
Level of treating places (Medical center as reference)				
Regional hospitals	1.11	1.03	1.19	0.004
Local hospitals	0.52	0.47	0.58	<0.001
Being treated at a public hospital	0.92	0.86	1.00	0.037
PECs (Charlson index = 0 as reference)				
Charlson index = 1	1.08	0.99	1.18	0.066
Charlson index = 2	1.52	1.34	1.72	<0.001
Charlson index > 2	2.50	2.16	2.90	<0.001
Mechanism (Fall on the same level as reference)				
Other mechanism	1.08	0.95	1.23	0.223
Car driver or passenger	1.09	0.86	1.39	0.483
Motorcycle rider or passenger	0.93	0.84	1.03	0.169
Pedal cyclist	0.93	0.73	1.18	0.540
Pedestrian hit by auto vehicle	1.60	1.36	1.89	<0.001
Traffic accident without clear mechanism	1.11	0.98	1.26	0.108
Fall from a height	1.58	1.38	1.81	<0.001
E-code missing	1.03	0.94	1.14	0.532
Anatomic injury (ICD/AIS < 3 as reference)				
Severe chest injury	3.41	3.07	3.79	<0.001
Severe Abdominal injury	3.41	2.76	4.21	<0.001
Severe lower limb	1.37	1.18	1.58	<0.001
Constant	0.05			<0.001

## References

[1] Department of Statistics, Ministry of Health and Welfare, Taiwan 2011 Statistics of Causes of Death. http://www.mohw.gov.tw/EN/Ministry/Statistic.aspx?f_list_no=474&fod_list_no=3485.

[2] Langlois J.A., Rutland-Brown W., Thomas K.E. (2006). Traumatic Brain Injury in the United States: Emergency Department Visits, Hospitalizations, and Deaths.

[3] Selassie A.W., Zaloshnja E., Langlois J.A., Miller T., Jones P., Steiner C. (2008). Incidence of long-term disability following traumatic brain injury hospitalization in the United States. J. Head Trauma Rehabil..

[4] Langlois J.A., Kegler S.R., Butler J.A., Gotsch K.E., Johnson R.L., Reichard A.A., Webb K.W., Coronado V.G., Selassie A.W., Thurman D.J. (2003). Traumatic brain injury related hospital discharges: Results from a 14-state surveillance system, 1997. MMWR Surveill. Summ..

[5] Coronado V.G., Xu L., Basavaraju S.V., McGuire L.C., Wald M.M., Faul M.D., Guzman B.R., Hemphill J.D., Centers for Disease Control and Prevention (CDC) (2011). Surveillance for traumatic brain injury-related deaths—United States, 1997–2007. MMWR Surveill. Summ..

[6] Chiu W.T., Hung C.C., Shih C.J. (1995). Epidemiology of head injury in rural Taiwan—A four year survey. J. Clin. Neurosci..

[7] Langlois J.A., Rutland-Brown W., Wald M.M. (2006). The epidemiology and impact of traumatic brain injury: A brief overview. J. Head Trauma Rehabil..

[8] Andersson E.H., Björklund R., Emanuelson I., Stålhammar D. (2003). Epidemiology of traumatic brain injury: A population based study in western Sweden. Acta Neurol. Scand..

[9] Baldo V., Marcolongo A., Floreani A., Majori S., Cristofolettil M., Dal Zotto A., Vazzoler G., Trivello R. (2003). Epidemiological aspect of traumatic brain injury in Northeast Italy. Eur. J. Epidemiol..

[10] Masson F., Vecsey J., Salmi L.R., Dartigues J.F., Erny P., Maurette P. (1997). Disability and handicap 5 years after a head injury: A population-based study. J. Clin. Epidemiol..

[11] Tagliaferri F., Compagnone C., Korsic M., Servadei F., Kraus J. (2006). A systematic review of brain injury epidemiology in Europe. Acta Neurochir. (Wien).

[12] Aenderl I., Gashaw T., Siebeck M., Mutschler W. (2014). Head injury—A neglected public health problem: A four-month prospective study at Jimma University Specialized Hospital, Ethiopia. Ethiop J. Health Sci..

[13] Andelic N.I., Anke A., Skandsen T., Sigurdardottir S., Sandhaug M., Ader T., Roe C. (2012). Incidence of hospital-admitted severe traumatic brain injury and in-hospital fatality in Norway: A national cohort study. Neuroepidemiology.

[14] Andriessen T.M., Horn J., Franschman G., van der Naalt J., Haitsma I., Jacobs B., Steyeberg E.W., Vos P.E. (2003). Epidemiology, severity classification, and outcome of moderate and severe traumatic brain injury: A prospective multicenter study. J. Neurotrauma.

[15] Annoni J.M., Beer S., Kesselring J. (1992). Severe traumatic brain injury—Epidemiology and outcome after 3 years. Disabil. Rehabil..

[16] Bruns J., Hauser W.A. (2003). The epidemiology of traumatic brain injury: A review. Epilepsia.

[17] Lee S.T., Lui T.N., Chang C.N., Wang D.J., Heimburger R.F., Fai H.D. (1990). Features of head injury in a developing country—Taiwan (1977–1987). J. Trauma.

[18] Masson F., Thicoipe M., Aye P., Mokni T., Senjean P., Schmitt V., Dessalles P.H., Cazauqade M., Labadens P., Aquitaine Group for Severe Brain Injuries Study (2001). Epidemiology of severe brain injuries: A prospective population-based study. J. Trauma.

[19] Chiu W.T., Kuo C.Y., Hung C.C., Chen M. (2000). The effect of the Taiwan motorcycle helmet use law on head injuries. Am. J. Public Health.

[20] Morris J.A., MacKenzie E.J., Edelstein S.L. (1990). The effect of preexisting conditions on mortality in trauma patients. JAMA.

[21] Charlson M.E., Pompei P., Ales K.L., MacKenzie C.R. (1987). A new method of classifying prognostic comorbidity in longitudinal studies: Development and validation. J. Chronic Dis..

[22] Baker S.P., O’Neill R., Haddon W., Long W.B. (1974). The injury severity score: A method for describing patients with multiple injuries and evaluating emergency care. J. Trauma..

[23] Linn S. (1995). The injury severity score—importance and uses. Ann. Epidemiol..

[24] MacKenzie E.J., Steinwachs D.M., Shankar B. (1989). Classifying trauma severity based on hospital discharge diagnoses. Validation of an ICD-9CM to AIS-85 conversion table. Med. Care.

[25] Durbin D.R., Localio A.R., MacKenzie E.J. (2001). Validation of the ICD/AIS MAP for pediatric use. Inj. Prev..

[26] Mullins R.J., Veum-Stone J., Helfand M., Zimmer-Gembeck M., Hedges J.R., Southard P.A., Trunkey D.D. (1994). Outcome of hospitalized injured patients after institution of a trauma system in an urban area. JAMA.

[27] Annest J.L., Fingerhut L.A., Gallagher S.S., Grossman D.C., Hedegaard H., Johnson R.L., Kohn M., Pickett D., Thomas K.E. (2008). Strategies to improve external cause-of-injury coding in state-based hospital discharge and emergency department data systems: Recommendations of the CDC workgroup for improvement of external cause-of-injury coding. MMWR Recomm. Rep..

[28] Koskinen S., Alaranta H. (2008). Traumatic brain injury in Finland 1991–2005: A nationwide register study of hospitalized and fatal TBI. Brain Inj..

[29] Tsai W.H., Chiu W.T., Chiou H.Y., Choy C.S., Hung C.C. (2004). Pediatric traumatic brain injuries in Taiwan: An 8-year study. J. Clin. Neurosci..

[30] (2018). American College of Surgeons. Advanced Trauma Life Support.

[31] Mullins R.J., Diggs B.S., Hedges J.R., Newgard C.D., Arthur M., Adams A.L., Veum-Stone J., Lenfesty B., Trunkey D.D. (2006). Regional differences in outcomes for hospitalized injured patients. J. Trauma.

[32] Singh G.K., Azuine R.E., Siahpush M., Kogan M.D. (2013). All-cause and cause-specific mortality among US youth: Socioeconomic and rural-urban disparities and international patterns. J. Urban Health.

